# Supplementary material for: No Such Thing as a Free-Rider? Understanding Drivers of Childhood and Adult Vaccination through a Multicountry Discrete Choice Experiment
Source: Vaccines (Basel). 2021 Mar 16;9(3):264. doi: 10.3390/vaccines9030264 (PMC7999942; doi:10.3390/vaccines9030264)
Supplement: Supplementary file 1 [file vaccines-09-00264-s001.zip › supplementary_material_S2_S3.pdf]

Supplementary Material for: "No such thing as a free-rider? Understanding drivers of childhood and adult vaccination through a multicountry discrete choice experiment"

Frederik Verelst[1*], Roselinde Kessels[2,3], Lander Willem[1], Philippe Beutels[1,4]

1 Centre for Health Economics Research and Modelling Infectious Diseases (CHERMID), Vaccine and Infectious Disease Institute (VAXINFECTIO), University of Antwerp, Antwerp, Belgium

2 Department of Data Analytics and Digitalization, Maastricht University, Maastricht, The Netherlands

3 Department of Economics, University of Antwerp, Antwerp, Belgium

4 School of Public Health and Community Medicine, The University of New South Wales, Sydney, Australia

*Corresponding author: Frederik Verelst, Centre for Health Economics and Modelling Infectious Diseases (CHERMID), Vaccine & Infectious Disease Institute (VAXINFECTIO), University of Antwerp, Campus Drie Eiken, Room S2.42, Universiteitsplein 1, 2610 Wilrijk (Antwerpen), Belgium, frederik.verelst@uantwerpen.be

Contents

Supplementary Material S2: PML model estimates including covariate interaction effects 2

Supplementary Material S3: Likert scale responses to vaccine attitude statements 14

Supplementary Material S2 : PML model estimates including covariate interaction effects

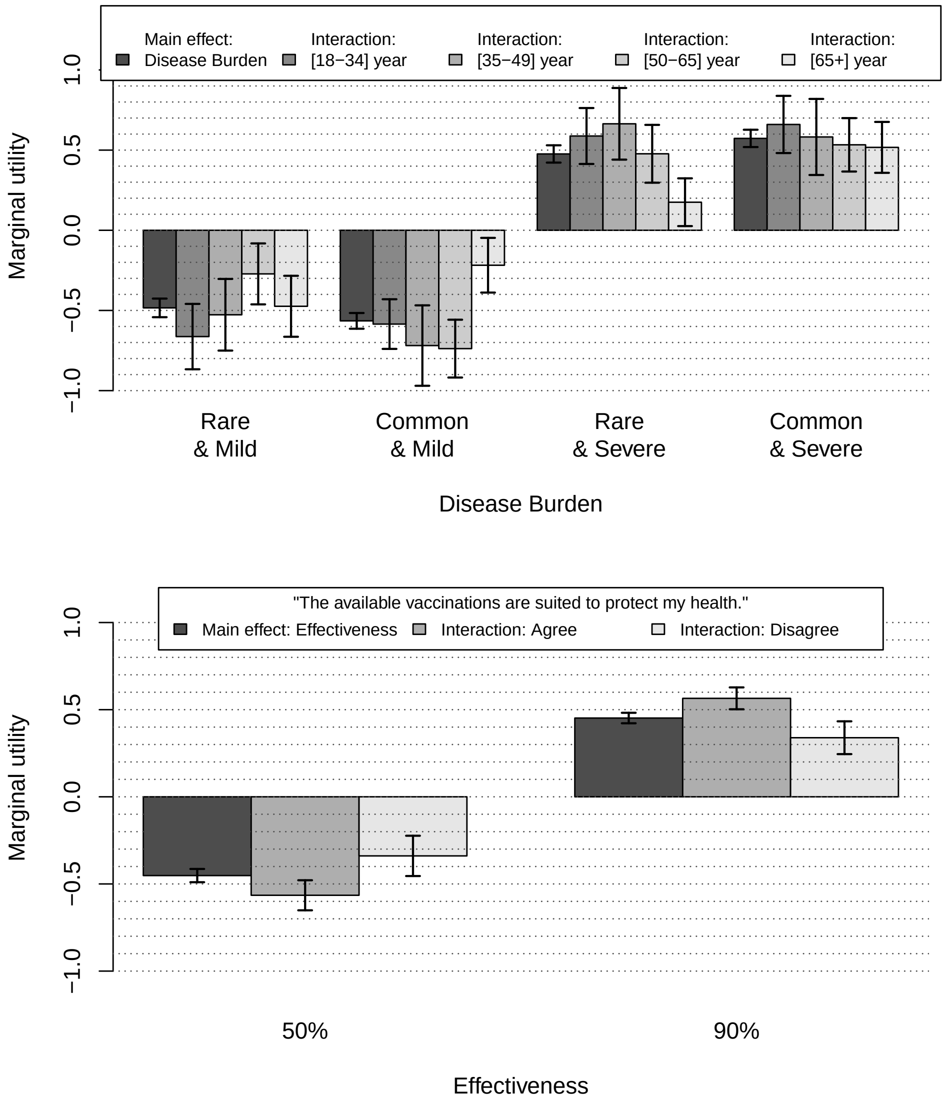

Figure S1 Marginal utilities for significant covariate interactions with disease burden (above) and vaccine effectiveness (below). 'Oneself' model, Belgium.

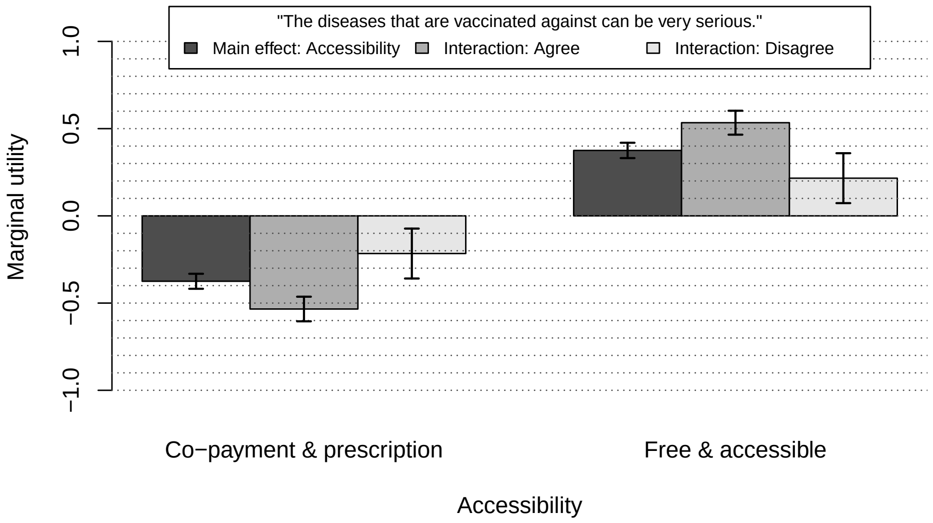

Figure S2 Marginal utilities for significant covariate interaction with accessibility. 'Youngest child' model, Belgium.

Table S1 Panel mixed logit model estimates (means and standard deviations) and significances of the attribute effects obtained from likelihood ratio (LR) tests. 'Oneself' model Belgium, including covariate interactions

Term	Mean estimate (std dev; subject std dev)	LR Chi-square	DF	P-value
**Accessibility**				
Co-payment & prescription	-0.433 (0.024; 0.376)	327.736	1	< 0.0001
Free & accessible	0.433 (0.022; 0.370)			
**Burden of disease**				
Rare & mild	-0.484 (0.058; 0.187)	257.965	3	< 0.0001
Common & mild	-0.565 (0.049; 0.132)			
Rare & severe	0.476 (0.054; 0.099)			
Common & severe	0.573 (0.054; 0.159)			
**Vaccine effectiveness**				
50%	-0.452 (0.038; 0.153)	192.235	1	< 0.0001
90%	0.452 (0.030; 0.144)			
**Population coverage (x10%)**	0.086 (0.009; 0.125)	66.439	1	< 0.0001
**Mild VRSE**				
Common	-0.179 (0.024; 0.112)	58.630	1	< 0.0001
Rare	0.179 (0.022; 0.101)			
**Vaccine effectiveness*Protective†**				
50%*agree	-0.113 (0.027; 0.162)	18.087	1	< 0.0001
50%*disagree	0.113 (0.028; 0.162)			
90%*agree	0.113 (0.025; 0.127)			
90%*disagree	-0.113 (0.024; 0.140)			
**Local coverage (x10%)**	0.049 (0.009; 0.084)	18.079	1	< 0.0001
**Burden of disease*Age**				
Rare & mild*[18-34]	-0.179 (0.087; 0.210)	33.439	9	0.0001
Rare & mild*[35-49]	-0.043 (0.096; 0.251)			
Rare & mild*[50-65]	0.212 (0.087; 0.254)			
Rare & mild*[65+]	0.010 (0.105; 0.269)			
Common & mild*[18-34]	-0.020 (0.081; 0.174)			
Common & mild*[35-49]	-0.154 (0.099; 0.223)			
Common & mild*[50-65]	-0.173 (0.087; 0.200)			
Common & mild*[65+]	0.347 (0.089; 0.246)			
Rare & severe*[18-34]	0.112 (0.085; 0.140)			
Rare & severe*[35-49]	0.188 (0.088; 0.146)			
Rare & severe*[50-65]	0.001 (0.079; 0.170)			
Rare & severe*[65+]	-0.301 (0.068; 0.135)			
Common & severe*[18-34]	0.087 (0.080; 0.146)			
Common & severe*[35-49]	0.009 (0.095; 0.174)			
Common & severe*[50-65]	-0.040 (0.079; 0.185)			
Common & severe*[65+]	-0.056 (0.076; 0.143)			

Note: Mean estimates corresponding to the last level of an attribute are calculated as minus the sum of the estimates for the other levels of the attribute. †Protective: "The available vaccinations are suited to protect my health."

Table S2 Panel mixed logit model estimates (means and standard deviations) and significances of the attribute effects obtained from likelihood ratio (LR) tests. 'Youngest child' model Belgium, including covariate interactions

Term	Mean estimate (std dev; subject std dev)	LR Chi-square	DF	P-value
**Vaccine effectiveness**				
50%	-0.584 (0.033; 0.238)	192.237	1	< 0.0001
90%	0.584 (0.041; 0.230)			
**Burden of disease**				
Rare & mild	-0.380 (0.063; 0.342)	163.220	3	< 0.0001
Common & mild	-0.621 (0.064; 0.458)			
Rare & severe	0.359 (0.054; 0.256)			
Common & severe	0.642 (0.068; 0.351)			
**Accessibility**				
Co-payment & prescription	-0.375 (0.043; 0.182)	94.231	1	< 0.0001
Free & accessible	0.375 (0.044; 0.177)			
**Population coverage (x10%)**	0.132 (0012.; 0.138)	93.690	1	< 0.0001
**Mild VRSE**				
Common	-0.229 (0.030; 0.140)	45.361	1	< 0.0001
Rare	0.229 (0.029; 0.134)			
**Local coverage (x10%)**	0.074 (0.014; 0.120)	27.390	1	< 0.0001
**Accessibility*Severity VPD†**				
Co-payment & prescription*agree	-0.159 (0.042; 0.178)	18.556	1	< 0.0001
Co-payment & prescription*disagree	0.159 (0.037; 0.179)			
Free & accessible*agree	0.159 (0.037; 0.185)			
Free & accessible*disagree	-0.159 (0.037; 0.175)			

Note: Mean estimates corresponding to the last level of an attribute are calculated as minus the sum of the estimates for the other levels of the attribute. †Severity VPD: "The diseases that are vaccinated against can be very serious."

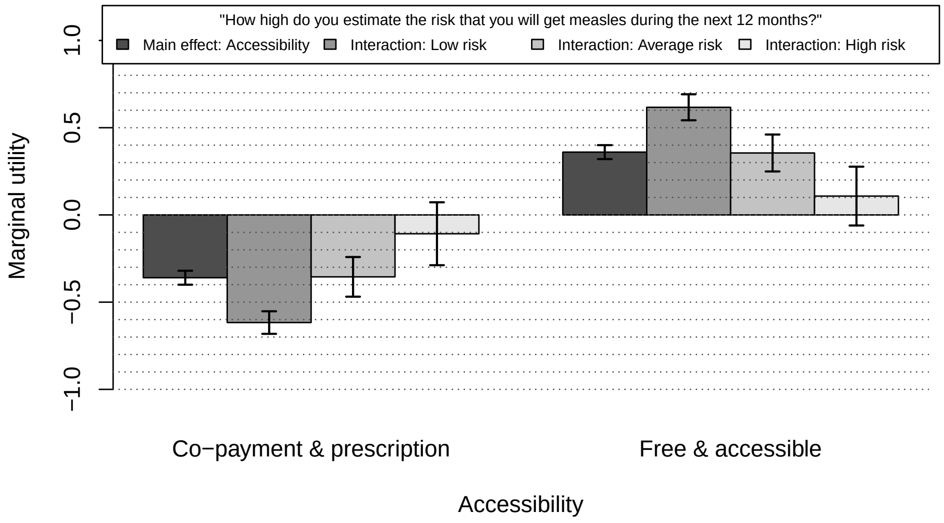

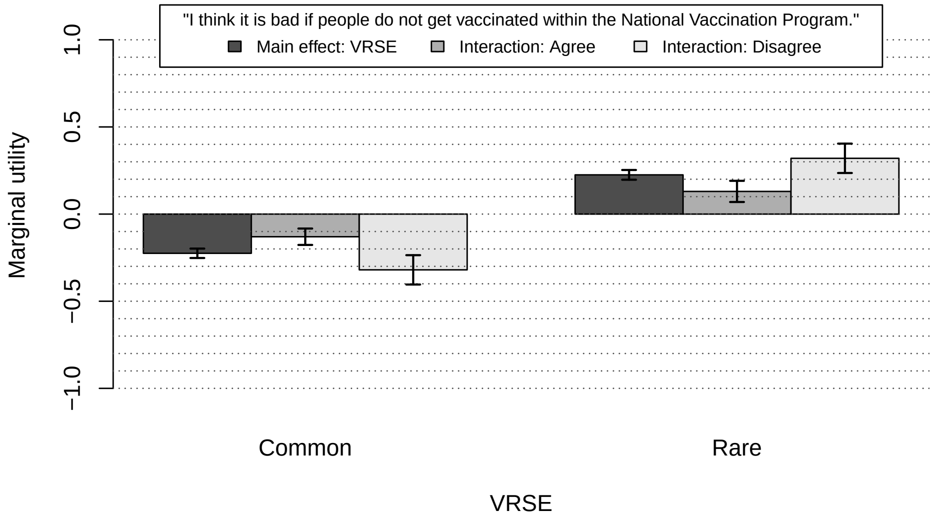

Figure S3 Marginal utilities for significant covariate interactions with accessibility (above) and VRSE (below). 'Oneself' model, United Kingdom.

Table S3 Panel mixed logit model estimates (means and standard deviations) and significances of the attribute effects obtained from likelihood ratio (LR) tests. 'Oneself' model United Kingdom, including covariate interactions

Term	Mean estimate (std dev; subject std dev)	LR Chi-square	DF	P-value
**Vaccine effectiveness**				
50%<br>90%	-0.720 (0.037; 0.301)<br>0.720 (0.034; 0.296)	431.561	1	< 0.0001
**Burden of disease**				
Rare & mild<br>Common & mild<br>Rare & severe<br>Common & severe	-0.545 (0.053; 0.310)<br>-0.368 (0.055; 0.438)<br>0.327 (0.046; 0.219)<br>0.586 (0.056; 0.259)	192.478	3	< 0.0001
**Accessibility**				
Co-payment & prescription<br>Free & accessible	-0.360 (0.040; 0.124)<br>0.360 (0.040; 0.120)	121.213	1	< 0.0001
**Population coverage (x10%)**	0.099 (0.010; 0.128)	94.070	1	< 0.0001
**Mild VRSE**				
Common<br>Rare	-0.225 (0.027; 0.087)<br>0.225 (0.028; 0.089)	66.328	1	< 0.0001
**Local coverage (x10%)**	0.084 (0.010; 0.080)	48.389	1	< 0.0001
**Accessibility*Measles susceptibility†**				
Co-payment & prescription*low risk<br>Co-payment & prescription*average risk<br>Co-payment & prescription*high risk<br>Free & accessible*low risk<br>Free & accessible*average risk<br>Free & accessible*high risk	-0.257 (0.042; 0.167)<br>0.005 (0.050; 0.143)<br>0.252 (0.067; 0.133)<br>0.257 (0.045; 0.168)<br>-0.005 (0.046; 0.129)<br>-0.252 (0.061; 0.134)	39.611	2	< 0.0001
**Mild VRSE*Bad if others don't vaccinate‡**				
Common*agree<br>Common*disagree<br>Rare*agree<br>Rare*disagree	0.095 (0.022; 0.091)<br>-0.095 (0.026; 0.093)<br>-0.095 (0.025; 0.088)<br>0.095 (0.025; 0.094)	17.408	1	< 0.0001

Note: Mean estimates corresponding to the last level of an attribute are calculated as minus the sum of the estimates for the other levels of the attribute. †Measles susceptibility: "How high do you estimate the risk that you will get measles during the next 12 months?" ‡Bad if others don't vaccinate: "I think it is bad if people do not get vaccinated within the National Vaccination Program."

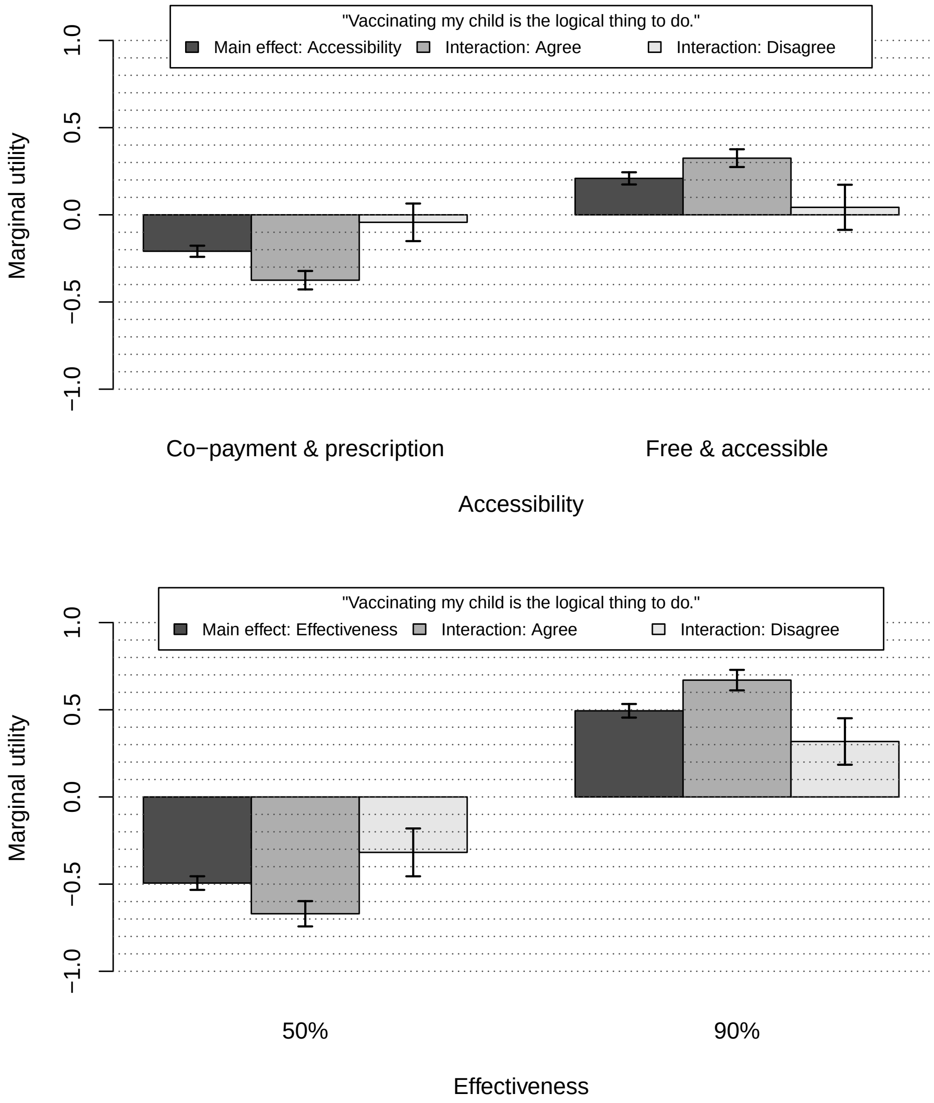

Figure S4 Marginal utilities for significant covariate interactions with accessibility (above) and vaccine effectiveness (below). 'Youngest child' model, United Kingdom.

Table S4 Panel mixed logit model estimates (means and standard deviations) and significances of the attribute effects obtained from likelihood ratio (LR) tests. 'Youngest child' model United Kingdom, including covariate interactions

Term	Mean estimate (std dev; subject std dev)	LR Chi-square	DF	P-value
**Vaccine effectiveness**				
50%<br>90%	-0.494 (0.039; 0.159)<br>0.494 (0.039; 0.152)	144.973	1	< 0.0001
**Population coverage (x10%)**	0.112 (0.010; 0.107)	95.334	1	< 0.0001
**Local coverage (x10%)**	0.100 (0.012; 0.100)	68.070	1	< 0.0001
**Burden of disease**				
Rare & mild<br>Common & mild<br>Rare & severe<br>Common & severe	-0.204 (0.048; 0.275)<br>-0.359 (0.046; 0.316)<br>0.198 (0.050; 0.174)<br>0.365 (0.046; 0.223)	71.456	3	< 0.0001
**Accessibility**				
Co-payment & prescription<br>Free & accessible	-0.209 (0.032; 0.140)<br>0.209 (0.035; 0.130)	40.391	1	< 0.0001
**Mild VRSE**				
Common<br>Rare	-0.146 (0.025; 0.094)<br>0.146 (0.023; 0.092)	31.056	1	< 0.0001
**Vaccine effectiveness*Logical†**				
50%*agree<br>50%*disagree<br>90%*agree<br>90%*disagree	-0.176 (0.036; 0.137)<br>0.176 (0.038; 0.130)<br>0.176 (0.036; 0.142)<br>-0.176 (0.036; 0.136)	26.399	1	< 0.0001
**Accessibility*Logical†**				
Co-payment & prescription*agree<br>Co-payment & prescription*disagree<br>Free & accessible*agree<br>Free & accessible*disagree	-0.166 (0.035; 0.132)<br>0.166 (0.030; 0.134)<br>0.166 (0.036; 0.136)<br>-0.166 (0.035; 0.127)	25.668	1	< 0.0001

Note: Mean estimates corresponding to the last level of an attribute are calculated as minus the sum of the estimates for the other levels of the attribute. †Logical: "Vaccinating my child is the logical thing to do."

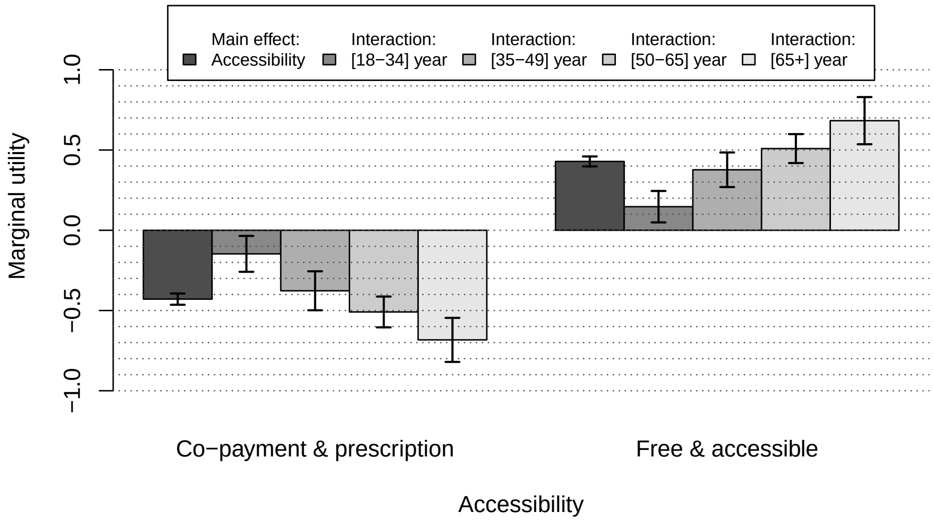

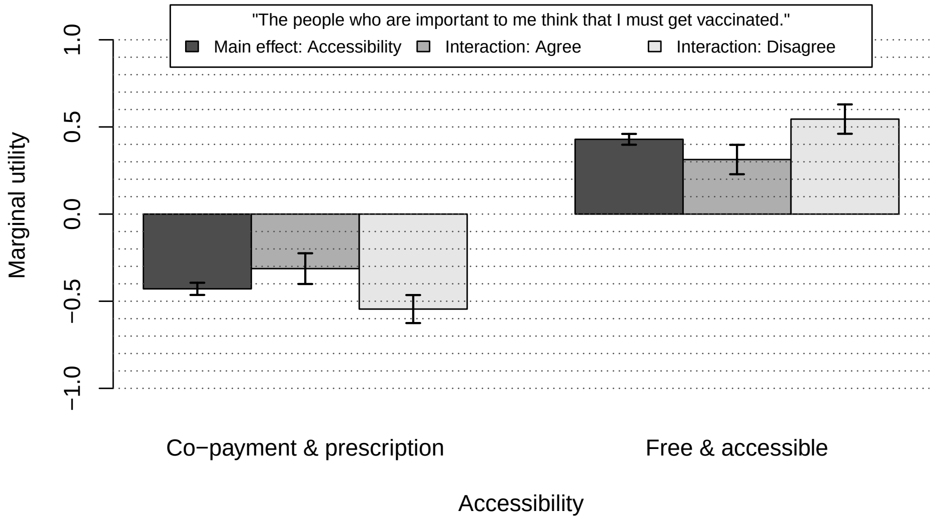

Figure S5 Marginal utilities for significant covariate interactions with accessibility (both charts). 'Oneself' model, France.

Table S5 Panel mixed logit model estimates (means and standard deviations) and significances of the attribute effects obtained from likelihood ratio (LR) tests. 'Oneself' model France, including covariate interactions

Term	Mean estimate (std dev; subject std dev)	LR Chi-square	DF	P-value
**Accessibility**				
Co-payment & prescription	-0.429 (0.035; 0.115)	215.395	1	< 0.0001
Free & accessible	0.429 (0.031; 0.106)			
**Vaccine effectiveness**				
50%	-0.403 (0.030; 0.237)	137.847	1	< 0.0001
90%	0.403 (0.031; 0.228)			
**Burden of disease**				
Rare & mild	-0.409 (0.051; 0.399)	125.353	3	< 0.0001
Common & mild	-0.358 (0.048; 0.455)			
Rare & severe	0.295 (0.050; 0.260)			
Common & severe	0.472 (0.052; 0.231)			
**Mild VRSE**				
Common	-0.184 (0.025; 0.097)	48.017	1	< 0.0001
Rare	0.184 (0.025; 0.102)			
**Population coverage (x10%)**	0.083 (0.011; 0.143)	47.914	1	< 0.0001
**Accessibility*Age**				
Co-payment & prescription*[18-34]	0.282 (0.060; 0.092)	42.015	3	< 0.0001
Co-payment & prescription*[35-49]	0.052 (0.052; 0.113)			
Co-payment & prescription*[50-65]	-0.080 (0.045; 0.159)			
Co-payment & prescription*[65+]	-0.254 (0.052; 0.157)			
Free & accessible*[18-34]	-0.282 (0.050; 0.092)			
Free & accessible*[35-49]	-0.052 (0.049; 0.120)			
Free & accessible*[50-65]	0.080 (0.050; 0.174)			
Free & accessible*[65+]	0.254 (0.058; 0.149)			
**Local coverage (x10%)**	0.069 (0.010; 0.098)	34.527	1	< 0.0001
**Accessibility*Peer influence†**				
Co-payment & prescription*agree	0.116 (0.026; 0.118)	27.529	1	< 0.0001
Co-payment & prescription*disagree	-0.116 (0.028; 0.109)			
Free & accessible*agree	-0.116 (0.028; 0.110)			
Free & accessible*disagree	0.116 (0.027; 0.107)			

Note: Mean estimates corresponding to the last level of an attribute are calculated as minus the sum of the estimates for the other levels of the attribute. †Peer influence: "The people who are important to me think that I must get vaccinated."

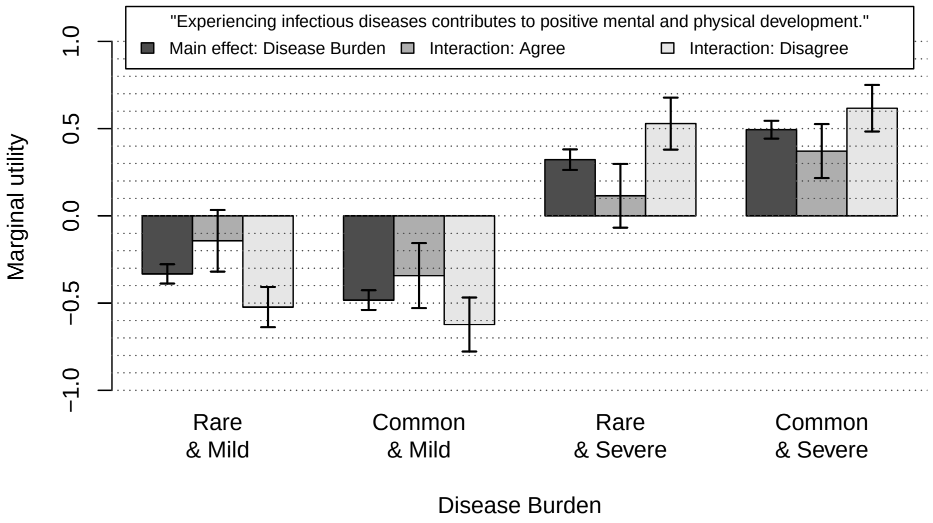

Figure S6 Marginal utilities for significant covariate interaction with burden of disease. 'Youngest child' model, France.

Table S6 Panel mixed logit model estimates (means and standard deviations) and significances of the attribute effects obtained from likelihood ratio (LR) tests. 'Youngest child' model France, including covariate interactions

Term	Mean estimate (std dev; subject std dev)	LR Chi-square	DF	P-value
**Vaccine effectiveness**				
50%<br>90%	-0.450 (0.028; 0.233)<br>0.450 (0.031; 0.273)	151.953	1	< 0.0001
**Accessibility**				
Co-payment & prescription<br>Free & accessible	-0.330 (0.024; 0.316)<br>0.333 (0.023; 0.296)	145.020	1	< 0.0001
**Burden of disease**				
Rare & mild<br>Common & mild<br>Rare & severe<br>Common & severe	-0.333 (0.055; 0.249)<br>-0.483 (0.056; 0.227)<br>0.322 (0.059; 0.178)<br>0.494 (0.051; 0.177)	124.816	3	< 0.0001
**Population coverage (x10%)**	0.092 (0.014; 0.090)	47.444	1	< 0.0001
**Mild VRSE**				
Common<br>Rare	-0.187 (0.024; 0.112)<br>0.187 (0.024; 0.104)	47.161	1	< 0.0001
**Local coverage (x10%)**	0.081 (0.011; 0.092)	45.150	1	< 0.0001
**Burden of disease*Positive development after infection†**				
Rare & mild*agree<br>Rare & mild*disagree<br>Common & mild*agree<br>Common & mild*disagree<br>Rare & severe*agree<br>Rare & severe*disagree<br>Common & severe*agree<br>Common & severe*disagree	0.190 (0.060; 0.216)<br>-0.190 (0.055; 0.167)<br>0.140 (0.058; 0.201)<br>-0.140 (0.057; 0.200)<br>-0.207 (0.055; 0.147)<br>0.207 (0.055; 0.164)<br>-0.123 (0.047; 0.158)<br>0.123 (0.056; 0.376)	27.754	3	< 0.0001
**Population coverage (x10%)*Confidence in vaccine info‡**				
Population coverage (x10%)*agree<br>Population coverage (x10%)*disagree	0.062 (0.014; 0.084)<br>-0.062 (0.013; 0.087)	19.992	1	< 0.0001

Note: Mean estimates corresponding to the last level of an attribute are calculated as minus the sum of the estimates for the other levels of the attribute. †Positive development after infection: "Experiencing infectious diseases contributes to positive mental and physical development." ‡Confidence in vaccine info: "I have confidence in the information about vaccinations that my care provider (*care provider is your GP or child healthcare professional/paediatrician) gives me."

Supplementary Material S3: Likert scale responses to vaccine attitude statements

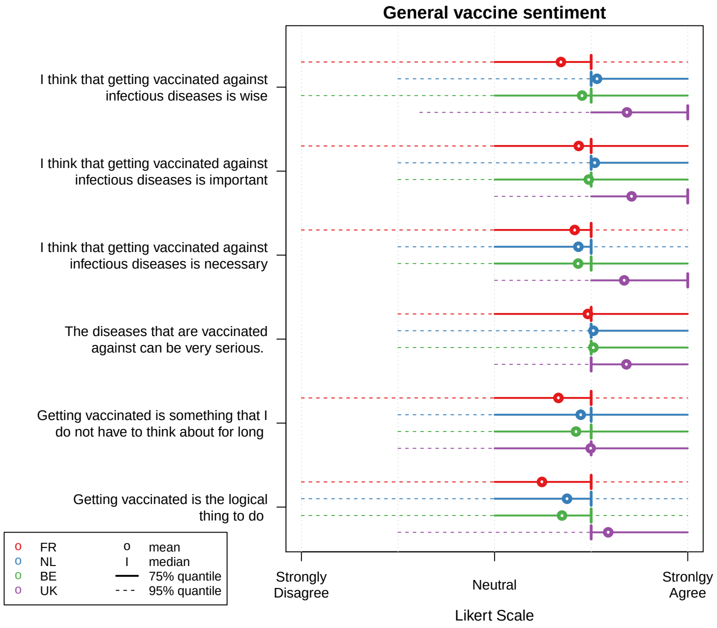

Figure S7 Likert scale responses to general vaccine statements in the 'oneself' group

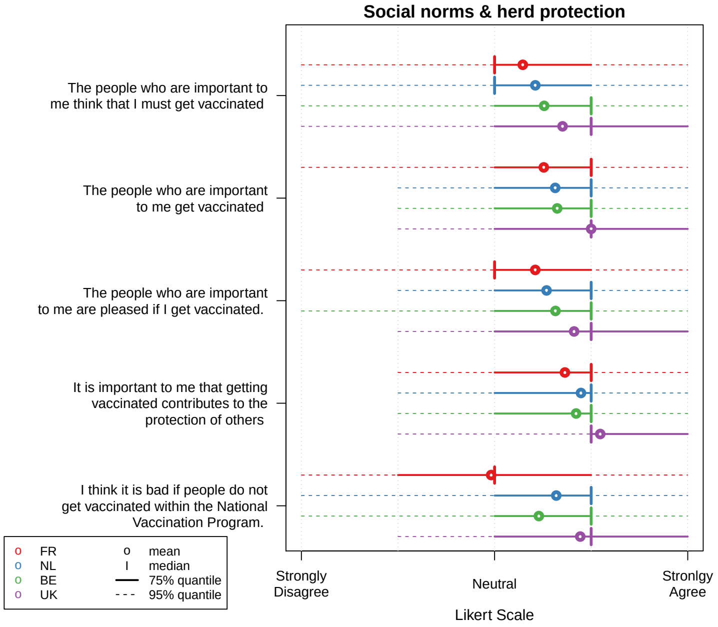

Figure S8 Likert scale responses to statements with respect to social norms and herd protection in the 'oneself' group

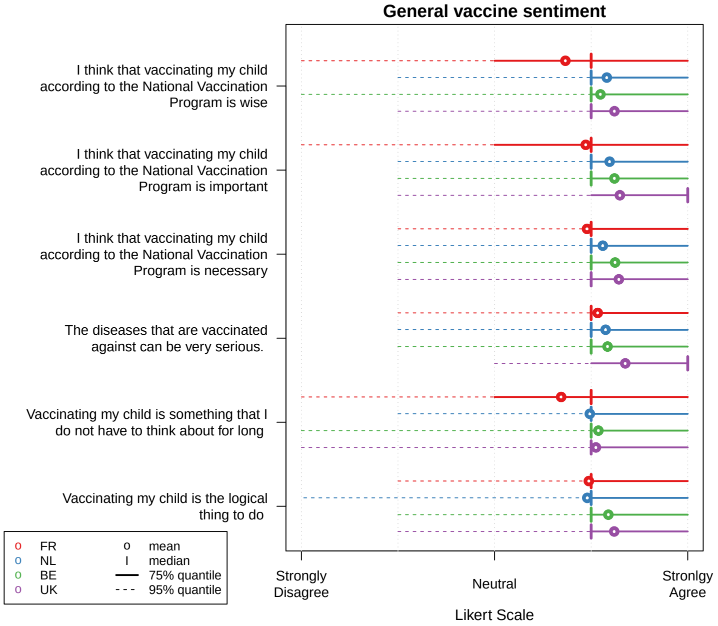

Figure S9 Likert scale responses to general vaccine statements in the 'youngest child' group

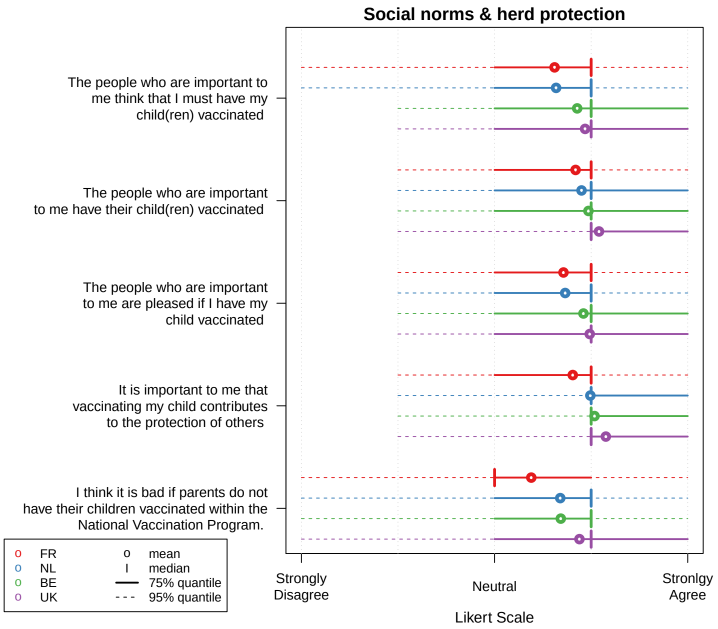

Figure S10 Likert scale responses to statements with respect to social norms and herd protection in the 'youngest child' group
